# Investigation of Liquid–Liquid Reaction Phenomena of Aluminum in Calcium Silicate Slag

**DOI:** 10.3390/ma17071466

**Published:** 2024-03-22

**Authors:** Harald G. R. Philipson, Maria Wallin, Kristian Etienne Einarsrud

**Affiliations:** Department of Materials Science and Engineering, Faculty of Natural Sciences, Norwegian University of Science and Technology (NTNU), 7034 Trondheim, Norway

**Keywords:** metal–slag interfacial reaction phenomena, aluminothermic, silicon, calcium aluminate slag

## Abstract

To achieve better process control of silicon (Si) alloy production using aluminum as a reductant of calcium silicate (CaO-SiO_2_) slag, it is necessary to understand the reaction phenomena concerning the behavior of formed phases at the metal-slag interface during conversion. The interfacial interaction behavior of non-agitated melt was investigated using the sessile drop method for varying time and temperature, followed by EPMA phase analysis at the vicinity of the metal–slag interface. The most remarkable features of the reaction were the accumulation of solid calcium aluminate product layers at the Al alloy–slag interface and spontaneous emulsion of Si-alloy droplets in the slag phase. The reduction is strictly limited at 1550 °C due to the slow transfer of calcium aluminates away from the metal-slag interface into the partially liquid bulk slag. Reduction was significantly improved at 1600–1650 °C despite an interfacial layer being present, where the conversion rate is most intense in the first minutes of the liquid–liquid contact. A high mass transfer rate across the interface was shown related to the apparent interfacial tension depression of the wetting droplet along with a significant perturbed interface and emulsion due to Kelvin–Helmholtz instability driven by built-up interfacial charge at the interface. The increased reaction rate observed from 1550 °C to 1600–1650 °C for the non-agitated melt was attributed to the advantageous physical properties of the slag phase, which can be further regulated by the stoichiometry of metal–slag interactions and the composition of the slag.

## 1. Introduction

The metallothermic reduction of calcium silicate slag (CaO-SiO_2_) using aluminum (Al) presents a promising alternative to the traditional submerged arc furnace (SAF) process for silicon (Si) metal production [1,2]. Industrially, the direct production of silicon by the reaction of Al alone with silicon dioxide (SiO_2_) is deemed impractical due to the inevitable formation of an inhibiting product layer of Al_2_O_3_ along the interface, which eventually halts the reaction [3,4]. To overcome this challenge, the introduction of calcium oxide (CaO) into the system serves as a fluxing agent. The CaO-SiO_2_ slag is engineered to serve as an optimal medium for liquid–liquid extraction and as a solvent for the resultant aluminum oxide (Al_2_O_3_). In particular, the controlled addition of CaO reduces the melting point and viscosity while increasing the surface tension of the slag melt [5,6].

This production method offers several benefits: (i) it can effectively utilize challenging industrial by-products and offers flexibility in raw material selection, thereby mitigating the risk of potential supply shortages; (ii) it operates at relatively low temperatures, resulting in reduced electricity consumption; (iii) by substituting the conventional carbon reductant with secondary Al, direct CO_2_ emissions are eliminated; and (iv) the process yields calcium aluminate (CaO-Al_2_O_3_) slag alongside Si, providing a valuable raw material for Al_2_O_3_ production. Recent pilot-scale studies [7,8], conducted under the European-funded SisAl Pilot project, have demonstrated the technical and economic viability of the aluminothermic reduction of calcium silicate slag, showcasing its potential as a multi-product production method for metallurgical-grade silicon (MG-Si) and high-alumina (Al_2_O_3_) slag.

Carbon, traditionally utilized as a reducing agent for SiO_2_ to produce Si, is replaced in this study by aluminum, employing simple chemical displacement reactions known as metallothermic reactions, which can be represented as the following:

R + MO → M + RO(1)

wherein the bonds between the metal (M) and oxygen (O) are broken, and the reductant (R) binds with oxygen to form oxide (RO). In the state-of-the-art process, in a liquid–liquid state, an arbitrary aluminum (Al) metal source reduces calcium silicate slag (CaO-SiO_2_), necessitating the establishment of equilibrium between the following reactions (Equations (2)–(4)):

(2) $$(4/3)\mathrm{Al}(l)+{\mathrm{SiO}}_{2}(s,\mathrm{cris}.)=(2/3){\mathrm{Al}}_{2}O_{3}(s,\mathrm{coru}.)+\mathrm{Si}(l),\;∆G_{1600{}^{\circ}C}^{0}\;=\;-140.2\;kJ$$

(3) $$(2/3)\mathrm{Al}(l)+\mathrm{CaO}(s,\mathrm{lime})=(1/3){\mathrm{Al}}_{2}O_{3}(s,\mathrm{coru}.)+\mathrm{Ca}(l),\;∆G_{1600{}^{\circ}C}^{0}\;=\;+75.3\;kJ$$

(4) $$(1/2)\mathrm{Si}(l)+\mathrm{CaO}(s,\mathrm{lime})=\mathrm{Ca}(l)+(1/2){\mathrm{SiO}}_{2}(s,\mathrm{cris}.),\;∆G_{1600{}^{\circ}C}^{0}\;=\;+145.4\;kJ$$

In this scenario, the slag comprises oxides, while the alloy contains the components. Under standard “stoichiometric” conditions (Al/SiO_2_ ratio) in laboratory and pilot-scale experiments, aluminum metal oxidizes to form Al_2_O_3_, and SiO_2_-CaO reduces to produce a silicon alloy alloyed with calcium and aluminum. Typically, this alloy contains weight percentages of 72–74% Si, 15–19% Ca, and 8–10% Al. The CaO-SiO_2_ slag, under these conditions, reduces to yield typical weight percentages of 48–52% Al_2_O_3_, 40–45% CaO, and 3–13% SiO_2_ [7,9]. The silicon alloy can undergo further refinement to produce metallurgical-grade silicon (MG-Si), while the slag can be subjected to hydrometallurgical treatment to produce either metallurgical-grade alumina (MGA) or high-purity alumina (HPA) [8,10,11,12,13,14,15].

The reduction of CaO by Al is a well-established phenomenon, as evidenced by prior investigations on the same system. For instance, prior studies have explored the reduction of solid pure CaO by liquid Al for Ca metal production [16,17], conducted under vacuum conditions. Additionally, the reduction of MnO-SiO_2_-CaO slag utilizing Al sources has been investigated [18], wherein SiO_2_ was completely reduced. The reduction of Ca is attributed to its low chemical activity within the metal phase coupled with its high activity within the slag phase. Consequently, an increased initial CaO content within the slag corresponds to an increased calcium content within the alloy.

Understanding the underlying interfacial reaction phenomena that may govern the reaction rate, reaction order, or liquid–liquid separation is crucial for addressing challenges related to process improvements and industrial upscaling. Previous investigations involving the same components have revealed that the reaction is rapid and approaches thermodynamic equilibrium under inductive stirring conditions in laboratory-scale experiments and under a combination of inductive stirring and gas purging in pilot-scale experiments. Studies examining the impact of varying Al-SiO_2_ stoichiometry and slag composition have resulted in fluctuations in both raw material yield and process stability, yet the mechanisms remain poorly understood [7,19,20]. In successful operations, the less dense Si alloy typically floats on top of the slag, albeit with varying degrees of metallic droplet entrainment in the slag phase. The presence of metallic entrainment in the slag diminishes the yield of tapped metal and can escalate the costs and complexity associated with hydrometallurgical slag treatment for the production of metallurgical-grade alumina (MGA) or high-purity alumina (HPA), necessitating additional cleaning steps [9,20].

Research into interfacial phenomena spans over a century, with increased interest in metal–slag interfacial phenomena following the seminal work of Kozakevitch et al. [21]. Davis and Rideal [22] provided a comprehensive review of three liquid–liquid interfacial phenomena, which can either depend on or be independent of each other [22,23]: (i) hydrodynamic phenomena, also discussed in terms of interfacial turbulence, arising from interfacial tension gradients at the interface due to Marangoni flow, (ii) a significant decrease in “dynamic” interfacial tension due to intense interfacial mass transfer, and (iii) the spontaneous formation of emulsions through “diffusion and stranding” or the entrainment of one phase into another, such as the dispersion of metal droplets in slag without the need for external mechanical agitation.

Extensive research on the interaction between liquid high-Al steel and liquid CaO-SiO_2_-Al_2_O_3_ slag has consistently demonstrated that the metallothermic reaction described by Equation (2) drives interfacial phenomena [20,23,24,25,26,27,28]. Observations and explanations of these interfacial phenomena are well documented, encompassing various metal–slag systems, such as Fe-Mn in contact with CaO-SiO_2_-MnO [26] and Cu-Ni-Al alloy with Li_2_O-Al_2_O_3_-SiO_2_ [29]. However, the relative significance of these phenomena in influencing overall reaction kinetics remains uncertain. To date, the extent to which overall kinetics can be elucidated or predicted by phenomena (i–iii) remains unclear.

Some researchers have examined metal–slag interfacial phenomena through the analysis of dynamic interfacial tension. Ooi et al. [25] highlighted the impact of increased aluminum content in iron as the driving force behind the reaction. Riboud and Lucas [23], along with Gaye et al. [5], demonstrated that intense mass transfer across the interface, quantified to be greater than 0.1 g oxygen m^−2^s^−1^ due to the high driving force, such as that of Equation (2), leads to a decrease in interfacial tension and the emulsification of metal droplets, provided that the reaction is not hindered by product accumulation at the interface.

Despite the complexities involved, research aligns on the assertion that dynamic interfacial tension correlates with interfacial reactions or fluid flow across the interface. Hydrodynamic phenomena are propelled by gradients of surface and interfacial tension along the interface between the contacting phases. These gradients arise from one or a combination of gradients in electrical potential (electrocapillary), temperature (thermocapillary), and/or surface-active solutes (solutocapillary). Richardson [30,31] attributed the decrease in metal–slag interfacial tension and the resulting interfacial turbulence, stemming from unequal local interfacial tensions, to electrocapillary effects. If the reaction rate is sufficiently high and the mass transfer rates of species differ across the metal–slag interface, such as the faster transfer of Al to the interface compared to Si from the interface, an interfacial charge develops. This electrostatic force augments London forces or dipole interactions across the interface, thereby decreasing interfacial tension. Richardson further posited that kinetics are either (i) facilitated by the flux of surface-active solutes, such as sulfur or oxygen in metallurgical reactions, across the interface due to a reaction, where fluctuations in interfacial tension induce interfacial turbulence, or (ii) surface-active elements reduce mass transfer coefficients by blocking diffusion or restricting the penetration of eddy currents. Sharan [28] expanded upon Richardson’s theory, proposing that the accumulation of surface-active oxygen at the interface induces dipole interactions and decreases the surface tension of iron, both of which contribute to the decrease in interfacial tension. Subsequently, Rhamdhani et al. [32] demonstrated that the electrocapillary effect predominated in the reaction described by Equation (2) between Fe alloy and CaO-SiO_2_-Al_2_O_3_ slag at 1600–1650 °C, accounting for 85% compared to the 15% attributable to the solutocapillary effect. They emphasized that the combined effects of all three mechanisms do not fully elucidate the interfacial phenomenon.

Chung and Cramb [12] delve into how the interface is disrupted by phenomenon (i) and identify it as the primary source initiating phenomenon (iii) (spontaneous emulsion) based on two fluid flow mechanisms at the interface: natural convection resulting from exothermic thermal energy release and Marangoni flow due to gradients in concentration, temperature, or potential along the interface. Marangoni flow induced by the reaction may lead to higher mass transfer rates from interfacial turbulence, thereby potentially accelerating the reaction. Owing to local variations in the magnitude of reaction and fluid flow along the interface, velocities parallel to the interface differ, giving rise to Kelvin–Helmholtz instability. A hallmark of this phenomenon is a perturbed or “wavy” interface, analogous to wind over the ocean of varying velocities that generates wave formation. Kelvin–Helmholtz instability increases at the metal–slag interface, eventually rendering the interface unstable and causing spontaneous emulsion. This process is driven by the minimization of surface area, with its magnitude being a function of interfacial differences (and viscosity) between the phases. Drawing from the Kelvin–Helmholtz model, Gopal proposed that the destabilization of the interface and consequently emulsion increase with the escalation of the reaction [27]. Bainbridge and Sawistowski [33] argued that the detachment of a droplet from one phase into another occurs after a necking stage governed by the Marangoni effect.

In many cases, the chemical reaction between a liquid metal and a liquid slag occurs rapidly, leading to the assumption that the reaction is governed by mass transfer, either within the metal or the slag, from or to the bulk/interface through a boundary layer, or through a product layer. As the occurrence of rapid reactions has been confirmed in prior studies [7,9], the examination of mass transfer within the metal and slag becomes of paramount importance. However, closer scrutiny of the slag is warranted due to potential reaction and mass transfer limitations. In the case of the alloy, the liquidus of associated phases shifts from aluminum to the Si-Al-Ca alloy that precipitates at significantly lower temperatures than the current process temperature range of interest (1550–1650 °C). Conversely, the CaO-SiO_2_-Al_2_O_3_ slag possesses a melting point closer to this temperature range, suggesting that solid phases could potentially precipitate if the slag conversion is not carefully controlled.

It is established that the primary conversion at the interface involves SiO_2_ to Si and Al to Al_2_O_3_, as described by Equation (2). This suggests that the formation of Al_2_O_3_ and adjacent phases such as CaAl_12_O_19_ and CaAl_4_O_7_ must be taken into account, given that the chemical reaction occurs more rapidly than the mass transport of the formed phases away from the interface. The likelihood of accumulated product increases if the initial metal–slag system is far from equilibrium and mass transfer is hindered by diffusion, potentially impeding overall mass transfer kinetics [29,34]. Although the viscosity of the slag can undergo significant changes during conversion, it generally remains lower for the metal phase compared to the slag phase. Furthermore, under the recently demonstrated raw material conditions [7,9,19,20], the slag-to-metal mass ratio ranges from 3 to 4, indicating that, intuitively, components in the slag must transfer across longer distances than their counterparts in the metal phase within an industrial furnace.

A common occurrence in liquid metal–slag melts is the phenomenon of interfacial saturation or the accumulation of a product layer at the metal–slag interface due to the faster reaction compared to the diffusion of the products. Numerous researchers have investigated the dissolution of solid Al_2_O_3_ in CaO-SiO_2_-Al_2_O_3_, including the dissolution of calcium aluminates [35,36]. It has been observed that mass transfer in the boundary layer or within the solid compound acts as the rate-limiting step for dissolution, particularly during periods when intermediate phases such as CaAl_4_O_7_ and CaAl_12_O_19_ are present [35]. Consequently, dissolution is contingent upon the viscosity of the slag and the diffusivity of Al_2_O_3_ (Al^3+^), both of which are influenced by the slag composition and temperature. Miao et al. [36] investigated the diffusion of CaAl_4_O_7_ inclusions in slag of composition 56.1(CaO)-38.6(Al_2_O_3_)-5.2(SiO_2_) (wt.%) and concluded that dissolution is limited by diffusion within the liquid slag phase at temperatures of 1550 °C and 1600 °C.

The objective of this study was to elucidate significant reaction phenomena occurring between pure Al metal and CaO-SiO_2_. The paper aims to substantiate metal–slag interfacial phenomena by investigating the impact of temperature and time on (i) flux behavior across the metal–slag interface, (ii) the formation of the interfacial oxide layer, and (iii) the formation and growth of Si alloy droplets.

The sessile drop method was deemed a suitable approach for several reasons. It allowed for the attainment of relatively stagnant flow conditions, minimizing the influence of external convection. Changes in the size of the wetting droplet were recorded and discussed in terms of the apparent alterations in the wetting behavior. To the best of the authors’ knowledge, there has been no previous study on the apparent interfacial interaction between pure Al and CaO-SiO_2_-Al_2_O_3_. Alongside qualitative and quantitative microscopic analyses of phases, reaction phenomena were thoroughly discussed.

The chosen Al metal-to-slag weight ratio and composition have yielded promising results in both laboratory and pilot-scale experiments, representing intermediate values within the spectrum of potential variations [20]. The reaction phenomena identified in this study are anticipated to be applicable across reasonable ranges of slag composition and reductant ratios.

## 2. Materials and Methods

The sessile drop experimental details are explained prior to the three parts describing the analytical methods that were used. In Part I, measured compositions of different phases and their locations were analyzed together with conversion degree with respect to temperatures 1550, 1600, and 1650 °C and times 3, 20, and 40 min. The phases were properly distinguished and quantified using an Electron Probe Micro Analyzer (EPMA) (EOL JXA-8500F, JEOL Ltd., Tokyo, Japan) and image analysis in ImageJ v0.5.7 [37]. In Part II, the in situ relative size change of the wetting droplet in the sessile drop furnace was discussed as a measure of apparent interfacial tension. Al alloy–slag interfacial interaction is combined with the conversion degree from Part I to assess the relationship between these. In Part III, the Si alloy droplets’ size and distribution were studied to assess whether a liberation, i.e., newly formed droplets, and coalescence effect can be derived from the identified principal phenomenon of droplet growth with respect to time–temperature variations.

### 2.1. Experimental

Pure aluminum (Al) was put on top of calcium-silicate slag powder at room temperature and heated to the three holding temperatures (1550, 1600, and 1650 °C) above the melting point of the slag and held at the temperatures for 3, 20, or 40 min. The weights of the Al and slag (Table 1) were on average 0.0245 ± 0.0028 g and 0.0870 ± 0.0097 g, respectively. The weights and composition were used to calculate the average stoichiometry to 0.977 ± 0.007 g based on the molar relationship of Equation (2). This chosen metal/slag ratio and the composition of reacting materials can be seen to represent the “middle values” of previous experiments [8,20] that have shown reactions with different stoichiometry and compositions of Al metal and slag. The stoichiometric value was subsequently input for thermodynamic equilibrium calculations in FactSage 8.1 [38]. The slag compounds CaO and SiO_2_ were pre-fused to calcium silicate slag (CaO-SiO_2_) by a project partner.

**Figure 1:**
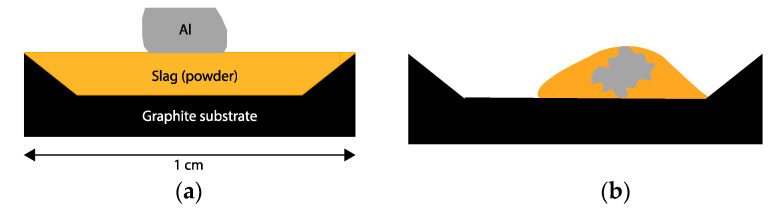
Cross section of experimental setup before heating (**a**) and after heating (**b**).

High-purity Al (99.999 wt.%) wire of diameter 2 mm was cut and grinded to the approximate stoichiometric weight using a SiC paper and placed on top of the dry slag of fine particle size (<71 µm) in a Ø-1 cm round dry graphite substrate (IG-88) of density 1.90 g/cm^3^ made by Titech (Shenzhen, China) (Figure 1a,b).

The sessile drop furnace was equipped with a C-type thermocouple and with a digital video camera (Allied Vision Prosilica GT2000, Edmund Optics, Inc., Barrington, IL, USA) with a telecentric lens (Navitar 1-50993D, Audio Video Supply, Inc. (AVS), San Diego, CA, USA) for recording images of molten samples with the resolution of 2048 × 1088 pixels at one frame per second. The furnace was vacuumed to between 10^−5^ and 10^−6^ mbar before it was filled with argon to overpressure (1030–1050 mbar) (0.1 L/min) and heated at 300 °C/min to 900 °C, followed by heating at 50 °C/min from 900 °C to the target temperatures (1550, 1600, and 1650 °C). Continuous logging of oxygen partial pressure was between log(p_O2_) = −15 and −17 at elevated temperature. Three parallel experiments of each time–temperature variation were conducted. Finally, the sample was quenched in argon (1 L/min) for rapid solidification at an average cooling rate of 500 °C/min from the target temperature to 1300 °C and 270 °C/min from 1300 °C to 1000 °C. The weight loss was calculated for every sample by subtracting the weight of the treated sample from the initial weight.

The solid sample piece of fused metal, slag, and graphite was cast in epoxy twice, firstly standing upright, and followed by cutting it in half and tilting it to have the reaction interface lying downward so the second epoxy could proceed. The solid epoxy of the reaction interface downwards was grinded and polished using the following grinding and polishing papers in chronologic order: SiC, largo, mol, and OP-U. The samples were then analyzed in EPMA.

### 2.2. Part I: Qualitative and Quantitative Analysis Using EPMA

The product sample analyzed by image analysis of the interface (phase mapping) gave colored images; Energy-Dispersive Spectrometry (EDS) of the Al alloy at the core and near the interface, where Al alloy denotes the alloyed Al metal after reaction; EDS of the slag next to the Al–slag interface and slag surrounding Si alloy droplets; and Wavelength-Dispersive X-ray Spectrometry (WDS) of the phase at the reaction barrier and phase surrounding the Si alloy droplets. Mapping at a magnification of 100 microns of the main elements (Al, Si, Ca, and O) was combined to sets of three elements producing four mapping images, AlSiCa, CaAlO, CaSiO, and SiAlO, allowing for the qualitative analysis of the overall metal and slag conversion. The compounds measured for the EDS of slag (and by default normalized) were Al_2_O_3_, SiO_2_, and CaO and minor MgO, MnO, TiO_2_, FeO, V_2_O_3_, CuO, NiO, ZnO, and SO_3_; and for the EDS of Al alloy, the elements were Al, Si, Ca, Mg, Mn, Ti, Fe, V, O, Cu, Ni, Zn, and S (and by default normalized).

The solid interfaces of the samples were analyzed using image analysis from colored mappings and compositions using EDS and WDS in EPMA for the different time–temperature variations. The areas of interest were (1) the composition of the Al alloy in the core and at the interface, (2) the composition, size, and distribution of coalesced Si alloy droplets, (3) the interfacial oxide product layer, and (4) the slag phase conversion of the slag near the Al alloy interface and in the bulk of the slag. The experimental data were evaluated with thermodynamic data.

#### 2.2.1. Method Determining the Average Composition of the Si Alloy Droplets in the Slag

The phase area fractions of the samples’ Si alloy droplets were normalized from quantified colors of the phases in EPMA mapping images. The %area Si_2_Al_2_Ca was adjusted for some of the mappings where this phase appeared in the Al alloy. The theoretical molar concentration of the present major phases Si, Si_2_Ca, and Si_2_Al_2_Ca, confirmed by WDS using EPMA in past studies [9,20], were converted to wt.% Si, Al, and Ca. This method assumes that area% = volume% = mass%. One or two mappings were conducted for every experiment and the average of the three parallels was plotted for each time–temperature condition.

#### 2.2.2. Analysis of Si Alloy Droplets in EPMA

The calculated mass per mapped area on the EPMA sample was assumed a measure of the conversion degree throughout this study. The mass was derived by assuming that the measured area represented perfect spheres and the density was weighted in accordance with composition. The result was then normalized to the total area measured to eliminate the effect of different appearances of the samples.

### 2.3. Part II: Melting Behavior of Wetting Droplet

The photographs taken every second during the sessile drop experiments were used for characterizing the behavior of the wetting droplet. The area of the part of the wetting droplet above the substrate was measured using ImageJ as will be shown in the results section. The measured areas were further analyzed as wetting droplet size or shrinkage.

### 2.4. Part III: Number and Radius of Si Alloy Droplets

The radius of the Si alloy droplets was derived from the measured areas in the EPMA images assuming perfect spherical particles. The data were used for distribution analysis of the time effect for the different holding temperatures. The data were manipulated in Python accordingly. Firstly, if parallels existed for the specific T-t case, the average counts and edges of parallel EPMA mappings were calculated using the Freedman–Diaconis rule for each parallel after ensuring common bin width. Secondly, the counts were normalized by the total area mapped for the Si alloy droplets. Thirdly, the process was repeated to calculate a new common bin width for parallel trials of same T-t condition and plotted to visually confirm that data had same bin width. Finally, the raw data were used to approximate a trend line based on a rolling average of 3 bins continuously.

### 2.5. Thermodynamic and Physical Property Calculations

Modules Equilibrium, Reaction, and Phase diagram in FactSage 8.1 [38] were used with databases FTlite and FToxid for the alloy and slag, respectively. The viscosity model in Factsage directly relates viscosity to the structure of the melt. The surface tension and density of the slags were calculated using Mills calculator [39]. The surface tension model is based on the partial molar approach and density calculations are based on the partial molar approach but with molar volumes of SiO_2_ and Al_2_O_3_ represented by equations [40].

## 3. Results

### 3.1. Part I

**Figure 2:**
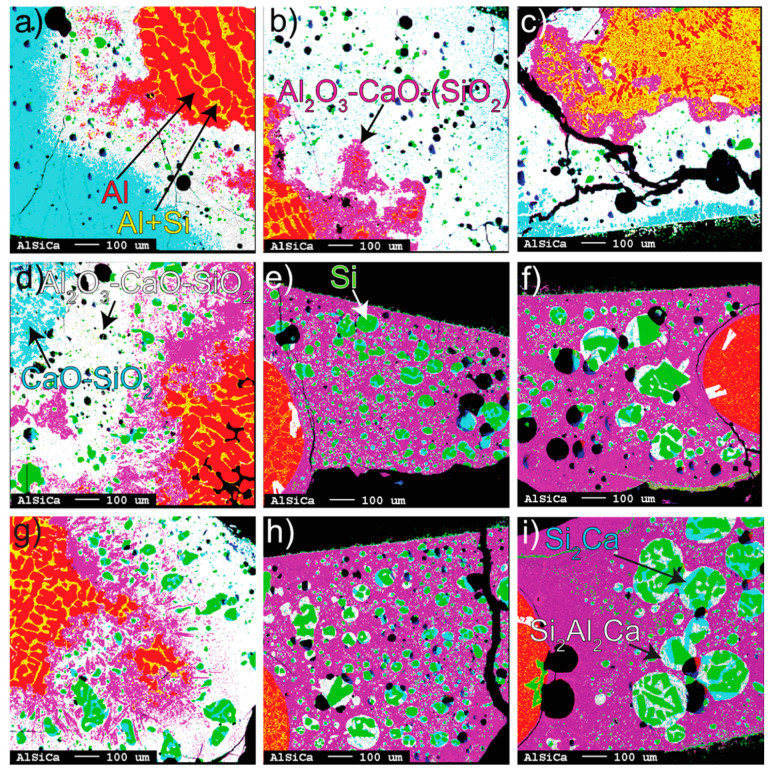
EPMA mappings. From left to right: (**a**–**c**) 3, 20, and 40 min at 1550 °C, (**d**–**f**) 3, 20, and 40 min at 1600 °C, (**g**–**i**) 3, 20, and 40 min at 1650 °C. All phases, with their corresponding colors, are identified by the arrows.

The conversion at 1550–1650 °C for 3 to 40 min can be seen in representative EPMA mapping images (Figure 2a–i) where the colors represent different metallic and oxide phases. The black represents either holes of removed Si alloy from polishing or cracks from cooling. The produced Si both diluted the initial Al (Al alloy) and formed to Si-alloy droplets that entrained within the slag. The size of the Si alloy droplets is relatively small at 1550 °C and less affected by time than the higher temperatures. In contrast, the coalescence of Si alloy droplets increases with time and temperature at 1600–1650 °C and changes from an irregular to spherical shape with time due to surface area minimization.

A gradient of mainly produced Al_2_O_3_ (purple), corresponding to calcium aluminates, was seen growing away from the Al alloy surface into the bulk slag. Purple represents close-to-equilibrium Al_2_O_3_-CaO-SiO_2_ slag in Figure 2e–i. At 1600–1650 °C, the calcium aluminates are completely or mostly dissolved to a homogenous slag between 3 and 20 min. Correspondingly, at 1550 °C, the dissolution was relatively slower. In the Al alloy, the phase Si_2_Al_2_Ca precipitated at irregular occurrences, although considered to a small degree with respect to the whole Al alloy (Figure 2e,f).

The Al alloy surface can be seen to change from perturbed or wavy at 1550 °C (all times) and the shortest holding time (3 min) at 1600–1650 °C to a smooth surface and spherical-shaped Al alloy corresponding to increased interfacial tension for longer times. This phenomenon is seen together with a relatively homogenous (purple) product slag. The detached part of the Al alloy was most frequent for 3 min, still with a surrounding calcium aluminate layer. Remarkably, for the partial conversion seen for 3 min at 1600–1650 °C, Si alloy droplets tend to locate in the slag region richer in SiO_2_ (white) and could indicate that significant Si was formed on the slag side of the Al alloy interface.

#### 3.1.1. Accumulation of Product Slag–Barrier at the Al Alloy–Slag Interface

**Figure 3:**
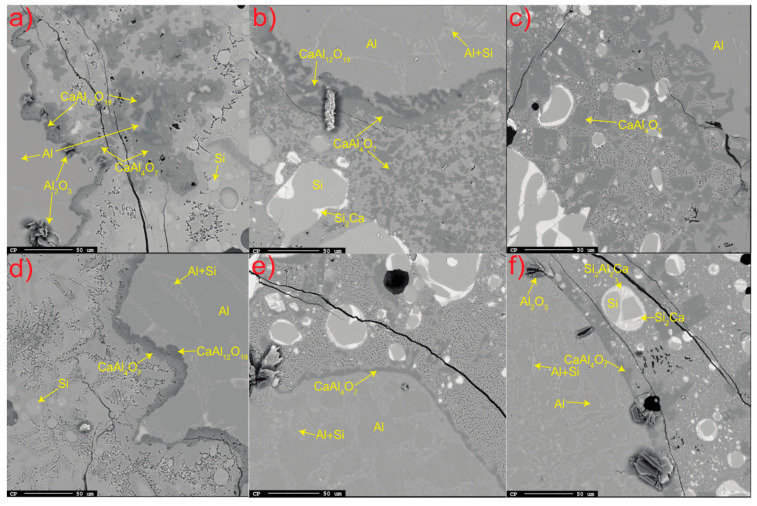
All significant phases at or near the Al alloy–slag interface; (**a**–**c**) 3 min at 1550–1650 °C and (**d**–**f**) 20 min at 1550–1650 °C. The alloy and slag are separated by the darker calcium aluminate layer.

In all trials, accumulated solid product layers of calcium aluminates were seen attached to the Al alloy surface and distributed as flakes away from the interface for the shortest holding time (Figure 3a–f). The product layer was observed to consist of CaAl_12_O_19_ closest to the Al metal followed by attached CaAl_4_O_7_ for all times at 1550 °C and for the 3 min holding time at 1600 °C. The distribution of CaAl_12_O_19_ at the Al alloy surface appeared more irregular than the more consistent layer of CaAl_4_O_7_. For the longer times at 1600–1650 °C, only CaAl_4_O_7_ was seen attached to the Al alloy surface and as relatively less dispersed flakes due to higher dissolution. At all temperatures, the clustered pure Al_2_O_3_ phase was seen at some parts at the interface.

**Figure 4:**
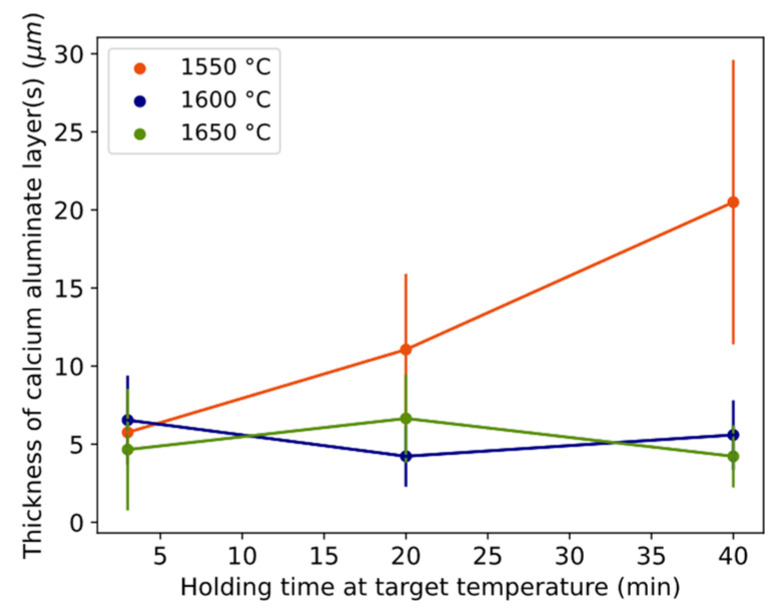
Thickness of the calcium aluminate layer(s) with time for different temperatures. Error bars equal one standard deviation. Length of whole error bar equals two standard deviations.

The thickness of the continuous and stable product layer(s) was measured for the different time–temperature conditions (Figure 4). Thickness tends to increase from 6 to 20 μm over time at 1550 °C, while it was a rather constant 4–7 μm at 1600–1650 °C.

#### 3.1.2. Conversion

**Figure 5:**
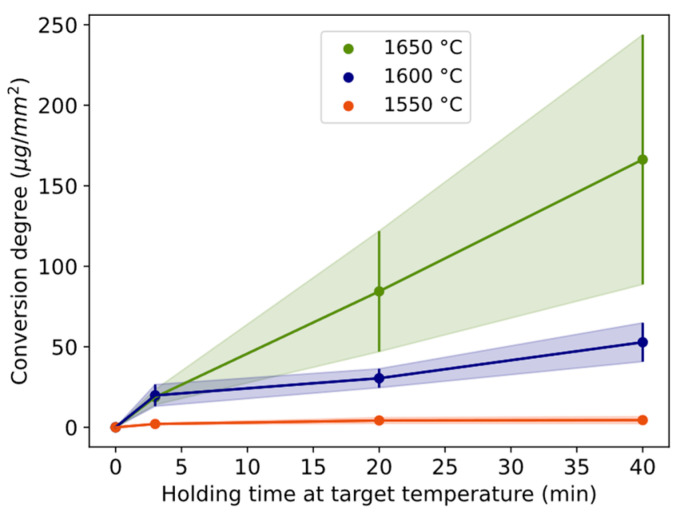
Conversion degree defined as mass of Si alloy droplets estimated per mapped area in EPMA, shown with holding times for different temperatures. Length of the whole error bar equals two standard deviations.

The estimated mass of Si alloy droplets per sample area mapped, as a measure of the conversion degree, is plotted for temperatures 1550–1650 °C for the 3, 20, and 40 min holding times (Figure 5 and Table 2). The trend is positive with both time and temperature. Comparing temperatures, only 3 min at 1600–1650 °C overlaps. At 1550 °C, the holding times have overlapping standard deviations. Correspondingly, they overlap at 1600 °C between 3 and 20 min and at 1650 °C between 20 and 40 min.

**Figure 6:**
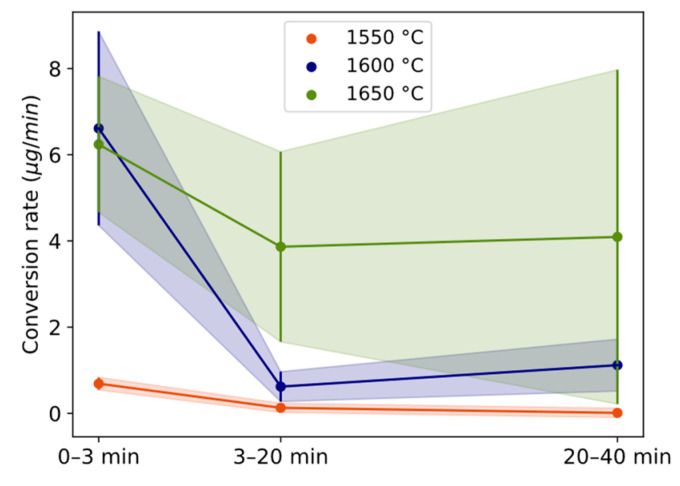
Conversion rate of Si alloy droplets with holding time for different temperatures. Length of whole error bar equals two standard deviations.

The conversion rate in terms of estimated micrograms of Si alloy is shown for three different time periods (0 to 3 min, 3 to 20 min, and 20 to 40 min) and the temperature of 1550–1650 °C in Figure 6. For all temperatures, the conversion rate is highest during the first 3 min (Figure 6) followed by a drastic decrease between 3 and 20 min. The range 3–20 min is statistically lower than 0–3 min for 1550 and 1600 °C. For 1600 and 1650 °C, the conversion rate increases after 20 min, although the data are not statistically significant between 3–20 and 20–40 min. The conversion degree (Figure 5) is higher for 1650 °C relative to 1600 °C because of the maintained higher rate after 3 min. The relative decrease in the reaction rate from 3 to 20 min for 1550 °C and 1600 °C is twice the decrease of 1650 °C.

#### 3.1.3. Al Alloy Composition

**Figure 7:**
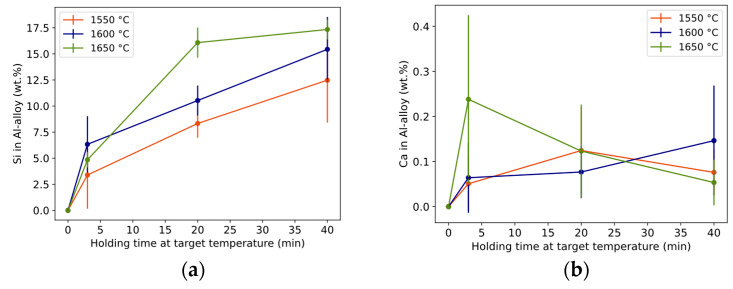
Composition of Si (**a**) and Ca (**b**) in the core of Al alloy (wt.% Al = 100 − wt.%Si). Length of whole error bar equals two standard deviations.

Si both diluted the initial Al metal into an Al alloy and dispersed as droplets in the slag phase. The initial Al metal can be seen increasingly alloyed with mainly Si (Figure 7a) and a small content of Ca (Figure 7b) for the given time–temperature conditions. The Si alloying trend is similar to the conversion degree in Figure 5. The holding time of 3 min is different from the longer times for 1550 °C and 1650 °C, and all times are different at 1600 °C. Si near the Al–slag interface was statistically similar to the Si content in the core of the Al alloy.

Ca concentration was much lower than Si in the Al alloy, in the range 0.05–0.24 wt.% and with an average of 0.11 wt.% for all trials (Figure 7b). However, the rare and randomly distributed Si_2_Al_2_Ca phase located at the Al-alloy surface contained some Ca. For all holding times and temperatures, the differentiation is statistically insignificant.

By assuming that the measured Al alloy compositions in Figure 7 were in contact with pure CaAl_4_O_7_, it was possible to calculate the equilibrium shown in Table 3. The measured Al composition corresponds well to the equilibrium of the Al alloy, i.e., the Al-alloy is in, or close to, equilibrium with the product layer. The measured Al content is somewhat lower, indicating that the product layer should have a slightly lower Al_2_O_3_/CaO ratio than the assumed CaAl_4_O_7_ (weight ratio 78.43/21.57). The Si and Ca content are similar and relatively deviating, respectively.

#### 3.1.4. Composition of Product Slag and Si Alloy Droplets

The concentrations in the product bulk slag in contact with the Si alloy droplets are shown in Table 4. A significant increase in the reduction of SiO_2_ and CaO occurs from 1550 °C to 1600 °C. The results at 1600 °C and 1650 °C correspond to results in published work [8,9,19,20], indicating that local equilibrium or partial equilibrium is established instantly at these temperatures. The only obvious overall trend with time was seen for 1550 °C, where Al_2_O_3_ tends to increase and SiO_2_ and CaO decrease.

The concentrations of slag components in the product bulk slag in contact with Si alloy droplets are presented as absolute values subtracted from the concentration of the slag right outside the calcium aluminates product layer (Table 5). All values for Al_2_O_3_ are positive and almost all are negative for SiO_2_ for all temperatures and times due to the higher reduction rate by Equation (2) at the Al alloy–slag interface than the mass transport away from/to the interface of the involved components. At 1550 °C, the gradients tend to reduce with time due to the relatively faster mass transport than reaction rate. The difference increases with time for 1600 °C (20 and 40 min) due to the higher conversion rate (Figure 6). The same relationship between the absolute wt.% difference and conversion rate is seen for 1650 °C.

At 1550 °C, the reduction of CaO at the Al alloy–slag interface occurs simultaneously as the supply of CaO from the bulk to the interface is slow. At the higher temperatures, the relationship is reversed, i.e., the mass transfer of CaO from the bulk is fast due to the dissolution of calcium aluminates and/or the reduction of CaO by Si alloy droplets is significant.

The calculated thermodynamic equilibrium in wt.% based on average (±std) Al/SiO_2_ stoichiometry in experiments, including impurities, can be seen in Table 6. The error represents standard deviations between upper, mean, and lower limits of the inserted sample’s stoichiometry.

The conversions of components in the product phases of Si alloy and slag relative to thermodynamic equilibrium (t.e.) are calculated accordingly:

(5) $$\mathrm{Conversion}\;\mathrm{of}\;\mathrm{component}\;i\;\mathrm{in}\;\mathrm{phase}\;j=\frac{\mathrm{measured}\;{\mathrm{wt}\%}_{i\;\mathrm{in}\;j\;}-\;\mathrm{initial}\;{\mathrm{wt}\%}_{i\;\mathrm{in}\;j}}{t.e.\;{\;\mathrm{wt}\%}_{i\;\mathrm{in}\;j\;}-\;\mathrm{initial}\;{\mathrm{wt}\%}_{i\;\mathrm{in}\;j}}$$

**Figure 8:**
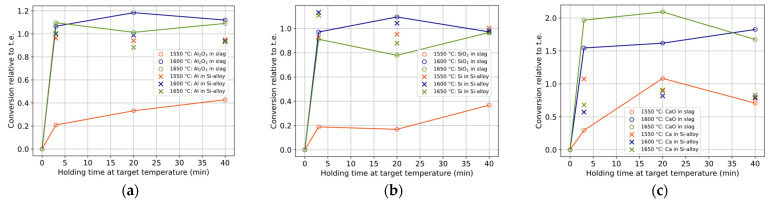
Conversions relative to t.e. of components (**a**) Al_2_O_3_ and Al, (**b**) SiO_2_ and Si, and (**c**) CaO and Ca in bulk slag and Si alloy, respectively. The colors and symbols represent temperatures and components, respectively.

The conversions for components Al and Al_2_O_3_ (a), Si and SiO_2_ (b), and Ca and CaO (c) are shown with holding times in Figure 8. A conversion larger than unity indicates more conversion than expected from the calculated thermodynamic equilibrium. For both the slag and alloy components, most of the conversions occur within 3 min, although they tend to increase with time at 1550 °C. In the first minutes of a reaction, a somewhat purer Si alloy relative to the thermodynamic equilibrium (t.e.) is produced due to the high Si compensated by a somewhat lower Ca (while Al is close to t.e. values). As the reaction proceeds, the impurity in the Si alloy seems to increase mainly due to increased Al content, even further away from t.e., which can be related to the increased activity of Al_2_O_3_ with the reaction. The shape of the curves (a) and (b) are similar, indicating that SiO_2_ replaces Al_2_O_3_, i.e., Equation (2). However, at 1600–1650 °C, the higher production of Al_2_O_3_ must also be explained by a relatively greater reduction of CaO by Al (Equation (3)) for the current conditions. The relatively higher converted CaO to that produced indicates Ca(g) evaporation. CaO shows the relatively largest difference between temperatures, with a statistically significant decrease with temperature for a fixed time (except overlapping at 40 min at 1600 and 1650 °C).

The following composition behavior can be seen for the Si alloy. Over time at 1550 °C, the Si and Al content tend to increase less, compared to the decrease in Ca. At 1600 and 1650 °C, the Si content tends to decrease, and Al and Ca tend to increase over time. Overall, only Si tends to get closer to the calculated thermodynamic equilibrium with time, while Ca and Al tend to converge towards a lower and higher value, respectively, the latter consistent with published research that reached close to, or reached, thermodynamic equilibrium [8,9,19,20].

### 3.2. Part II

#### In Situ Analysis of Al Metal and Slag Melting

**Figure 9:**
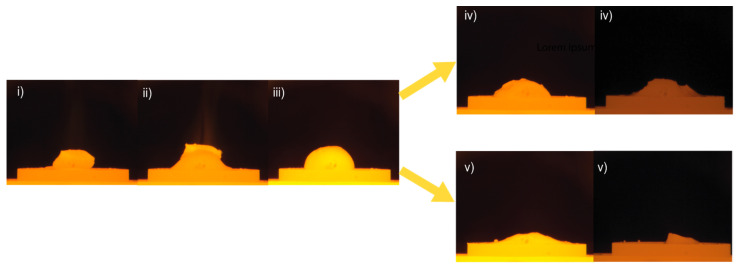
Wetting behavior of the metal and slag separated into different characteristic sequences: (**i**) Al keeps its initial shape well above its melting point; (**ii**) slag starts to melt at 1466 ± 4.6 °C and starts to “climb” the Al; (**iii**) wetted droplet consolidates at 1534 ± 9.6 °C and in 1.4 ± 0.2 min after the slag melts; (**iv**) at 1550 °C for 3, 20, and 40 min and 1600–1650 °C for 3 min: the droplet deteriorates to an irregular shape and the flattening/shrinkage ends, after holding for 3.0 ± 1.7 min at 1550 °C; (**v**) at 1600–1650 °C for 20 and 40 min: droplet slowly flattens out with time. Flattening starts at 3.8 ± 0.6 min and 2.9 ± 1.5 min of the holding time 1600 and 1650 °C, respectively.

**Figure 10:**
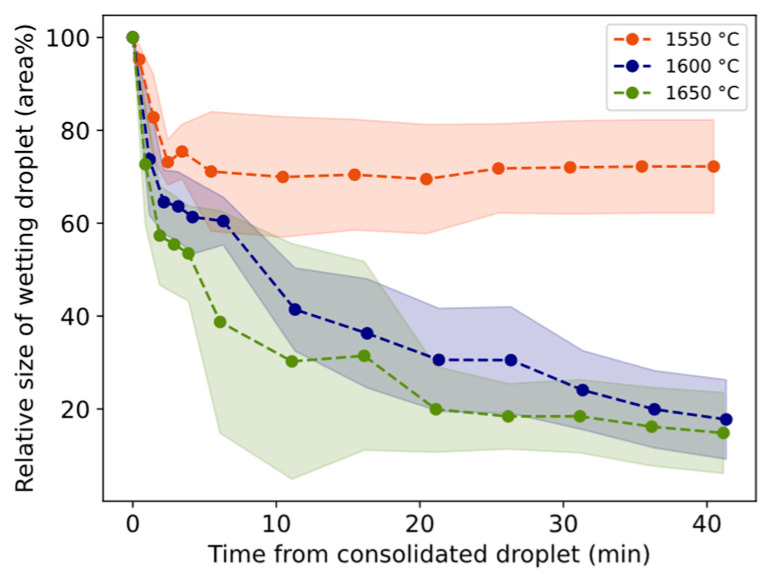
Relative size of wetting droplet. Whole vertical length of lighter color equals two standard deviations.

The wetting process can be viewed in four sequences, depicted in Figure 9. The wetting behavior was very similar for all experiments in sequences (i)–(iii). In sequence (i), the Al piece is kept intact, although the temperature is hundreds of degrees above its melting point prior to sequence (ii), as the slag rapidly melted and began to “climb” or spread over the Al. Only during this “climbing” sequence was an intense radiance (visible as a beam of light) seen, a potential effect of an exothermic reaction. In sequence (iii), the Al and slag had completely consolidated as a round droplet. The droplet either deteriorated to a somewhat steady irregular shape (iv) or slowly flattened out with time (v) depending on holding time and temperature, as can be seen in Figure 10.

In sequence (v), the collapse continued until the sample was, or was close to, completely flattened for trials at 1600 °C with a 40 min holding time and at 1650 °C during the 20 and 40 min holding times. The wetting between Al and the slag was more significant at 1650 °C indicated by the clearer “floating” of the Al on top of the slag in the end of sequence (ii). For the lower temperatures, the Al tended to “sink” into the slag more easily.

**Figure 11:**
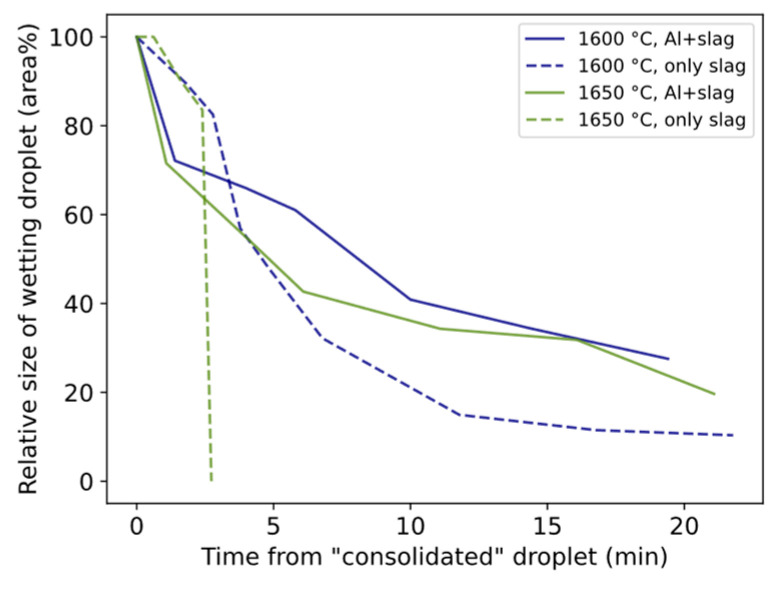
Comparison between relative size of wetting droplet for Al + slag and only slag.

The relative size of the wetting droplet is in general smaller for 1550 °C than for 1600–1650 °C (Figure 10). In two trials, slag only was heated to 1600 °C and 1650 °C and held for 20 min. The relative size change is shown in Figure 11 together with corresponding trials containing both slag and Al. For both temperatures, the droplet size decreases slower during the first minutes followed by a sharper decrease for longer times compared to Al + slag trials.

### 3.3. Part III

**Figure 12:**
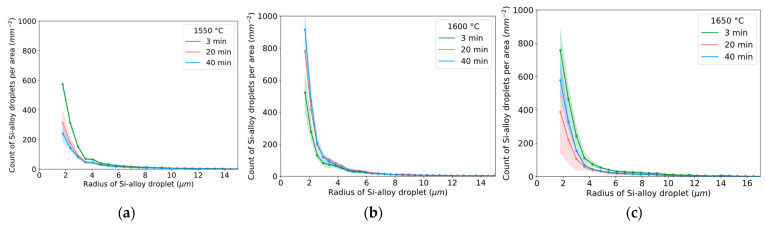
Radius distribution for all measured particles at temperatures of (**a**) 1550 °C, (**b**) 1600 °C, and (**c**) 1650 °C for all holding times. Whole vertical length of lighter color equal two standard deviations.

The estimated radius distribution of Si alloy droplets for different times and temperatures is shown in Figure 12a–c. The characteristic shape of the curve indicates that a relatively high number of small droplets exist, decreasing with an increased radius from approximately 2 to 4 μm. A similar number of small droplets for 3 min was seen for 1550–1600 °C. At 1650 °C, a higher number of small droplets were seen. Overall, the particle size and distribution are different between 3 min and 20/40 min for all temperatures, indicating the kinetic effect on droplet liberation and coalescence.

**Figure 13:**
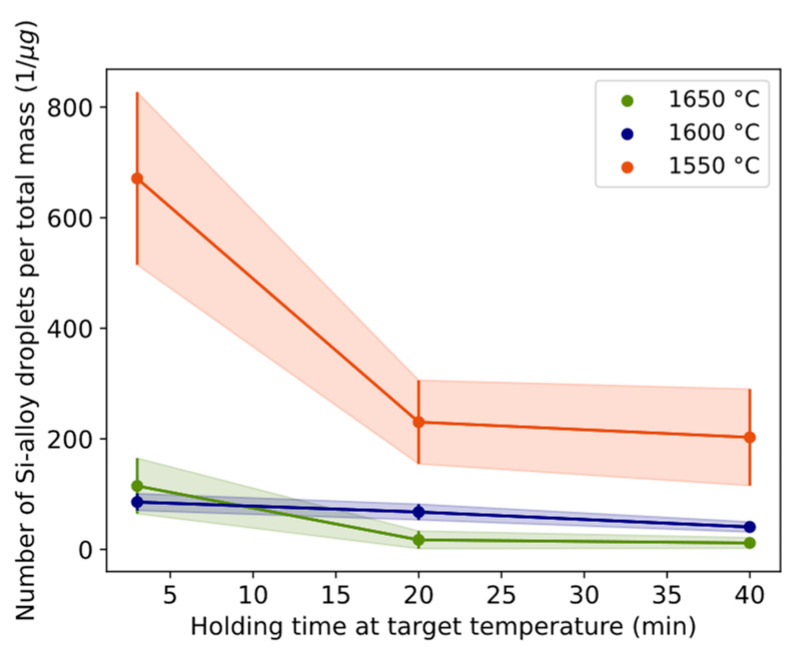
Relationship between number per mass of Si alloy droplets and holding time for different temperatures. Whole vertical length of error bars equals two standard deviations.

Another indication of the coalescence behavior can be shown by excluding the effect of conversion by calculating the number of Si alloy droplets per total mass with time in Figure 13. A smaller number of droplets per total mass should indicate more coalesced droplets. The largest change (85%) was between 3 and 20 min for 1650 °C followed by the change (67%) at 1550 °C for the same time range. Assuming the slope corresponds to the coalescence rate, the calculated results are shown in Table 7. A positive value indicates a relatively higher liberation rate than coalescence, which occurs during the first three minutes. The reaction is initially driven by liberation predominating over coalescence. After 3 min and over time, the predominance of the coalescence rate over the liberation rate tends to decrease (during 3–20 min) and stabilize (during 20–40 min).

## 4. Discussion

As only a partial reaction between the Al and slag occurred, the results stand out from past studies [8,9,19] where global thermodynamic equilibrium, or partial equilibrium, was reached by the complete reduction of Al metal to a bulk Si alloy. Here, the steady-state or stagnant flow condition led to less reduction as no external convection is contributing to convection, which facilitated a discussion on the fundamental conversion path at the metal–slag interface. The conversion was characterized by the dispersion of Si alloy droplets in the slag phase and Si alloying the Al metal to an Al alloy. The shrinking of the Al metal/Al alloy, wavy interface, and Si alloy emulsion increase the specific surface area, which in turn promotes the global reaction rate. Another novel characteristic of reaction was the product layer on the Al alloy surface, consisting of solid calcium aluminates CaAl_4_O_7_ and CaAl_12_O_19_. These phenomena are further discussed.

### 4.1. Dissolution Mechanism

As depicted in Figure 6, the conversion rate exhibits its most rapid increase during the initial 3 min, attributed to the elevated driving force for the reaction during this period, followed by a slowed reaction rate with time. Mostly elemental Si is formed initially, seen by the composition of the Al alloy (Figure 7) and Si alloy droplets (Figure 8), i.e., the reaction in Equation (2) is predominant. Combined with the slow transport of the slag product, the accumulation and formation of solid product layers of CaAl_4_O_7_ and CaAl_12_O_19_ are formed. These phases hence accumulate at the Al–slag interface as SiO_2_ is consumed at a higher rate than the corresponding dissolution rate or the reaction rate of the CaO-containing calcium aluminates. CaAl_2_O_4_ was seen as one of the major phases in the bulk slag in agreement with the expectation from the rapid solidification of the equilibrium slag composition. The lower melting point of CaAl_2_O_4_ at 1596 °C [38] and high diffusion coefficient explain that this phase was not noticeably seen as an attached layer at the Al alloy interface at any temperatures at 1550–1650 °C, although it could have been solid, or partially solid, at 1550 °C. However, a liquid gradient for 3 min at 1600–1650 °C representing a locally relatively higher Al_2_O_3_/CaO ratio than expected by t.e. explains that the solidified Al_2_O_3_ gradient decreases with increased distance perpendicular to the interface (Figure 2). The formation of CaAl_4_O_7_(s) and CaAl_12_O_19_(s) is suggested to occur due to the high driving force for the reaction according to the overall reactions:

(6) $$12\mathrm{Al}(l)+9{\mathrm{SiO}}_{2}(l)+\mathrm{CaO}(l)=9\mathrm{Si}(l)+{\mathrm{CaAl}}_{12}O_{19}(s),\;∆G_{1600{}^{\circ}C}^{0}\;=\;-152.9\;kJ$$

(7) $$4/3\mathrm{Al}(l)+{\mathrm{SiO}}_{2}(l)+1/3\mathrm{CaO}(l)=\mathrm{Si}(l)+1/3{\mathrm{CaAl}}_{4}O_{7}(s),\;∆G_{1600{}^{\circ}C}^{0}\;=\;-174.6\;kJ$$

which can be simplified to Equation (2) (4Al + 3SiO_2_ = 2Al_2_O_3_ + 3Si), that is, CaO does not initially take part in the metallothermic reaction and solid products are formed from the liquid–liquid reaction. The phases CaAl_4_O_7_ (78.4-wt.% Al_2_O_3_ and 21.6-wt.% CaO) and CaAl_12_O_19_ (91.6-wt.% Al_2_O_3_ and 8.4-wt.% CaO) have melting points of 1754 °C and 1833 °C, respectively [38]. This means that for reaction Equation (7) to occur, Al or SiO_2_ must diffuse through the solid product layer of CaAl_12_O_19_(s), as the inner layer consists of CaAl_12_O_19_(s), as seen in Figure 3. The reactions in Equations (6) and (7) assume that the following reactions, Equations (8) and (9), are fast and occur simultaneously:

(8) $$6{\mathrm{Al}}_{2}O_{3}(s,\mathrm{coru}.)+\mathrm{CaO}(l)={\mathrm{CaAl}}_{12}O_{19}(s),\;∆G_{1600{}^{\circ}C}^{0}\;=\;-109.4\;kJ$$

(9) $$2{\mathrm{Al}}_{2}O_{3}(s,\mathrm{coru}.)+\mathrm{CaO}(l)={\mathrm{CaAl}}_{4}O_{7}(s),\;∆G_{1600{}^{\circ}C}^{0}\;=\;-101.4\;kJ$$

Studies have shown that these phases form as an intermediate product during the dissolution of Al_2_O_3_(s) in contact with the equivalent product slag system as in the present study [35,41]. Dissolution reactions of the calcium aluminates also occur as shown in Equations (10) and (11), for the former equation given that Ca^2+^ cations (CaO) diffuse through the CaAl_4_O_7_(s) layer.

(10) $$1/3{\mathrm{CaAl}}_{12}O_{19}(s)+2/3\mathrm{CaO}(l)={\mathrm{CaAl}}_{4}O_{7}\;(s),\;∆G_{1600{}^{\circ}C}^{0}\;=\;-65.0\;kJ$$

(11) $${\mathrm{CaAl}}_{4}O_{7}(s)+\mathrm{CaO}(l)=2{\mathrm{CaAl}}_{2}O_{4},\;∆G_{1600{}^{\circ}C}^{0}\;=\;-62.2\;kJ$$

The diffusion coefficient of Ca^2+^ cations, due to their smaller size, have been reported as higher than the ${\mathrm{AlO}}_{x}^{y-}$ anion complex (simplified to Al_2_O_3_) [36]. The observed dendrite-like growth of CaAl_12_O_19_ on the Al alloy side suggests diffusion of the Ca^2+^ cation through the CaAl_4_O_7_ layer; therefore, the higher flux of Ca^2+^ is not rate-controlling. Once the activity (or supply) of SiO_2_ (or Si ions) is low due to reaction Equations (6) and (7), the layers can continue to grow by reaction between Al and liquid Al_2_O_3_-CaO (Equations (12)–(14)) to produce Ca(l); however, this requires diffusion through the layer(s). The driving force for Equation (12) is highest, given that the supply of Al_2_O_3_ and CaO has a high Al_2_O_3_/CaO ratio.

(12) $$1.33\mathrm{Al}(l)+5\mathrm{CaO}(l)+17.33{\mathrm{Al}}_{2}O_{3}(l)=2\mathrm{Ca}(l)+3{\mathrm{CaAl}}_{12}O_{19}(s),\;∆G_{1600{}^{\circ}C}^{0}\;=\;-627.7\;kJ$$

(13) $$1.33\mathrm{Al}(l)+5\mathrm{CaO}(l)+5.33{\mathrm{Al}}_{2}O_{3}(l)=2\mathrm{Ca}(l)+3{\mathrm{CaAl}}_{4}O_{7}(s),\;∆G_{1600{}^{\circ}C}^{0}\;=\;-329.7\;kJ$$

(14) $$1.33\mathrm{Al}(l)+5\mathrm{CaO}(l)+2.33{\mathrm{Al}}_{2}O_{3}(l)=2\mathrm{Ca}(l)+3{\mathrm{CaAl}}_{2}O_{4}(s),\;∆G_{1600{}^{\circ}C}^{0}\;=\;-202.5\;kJ$$

**Figure 14:**
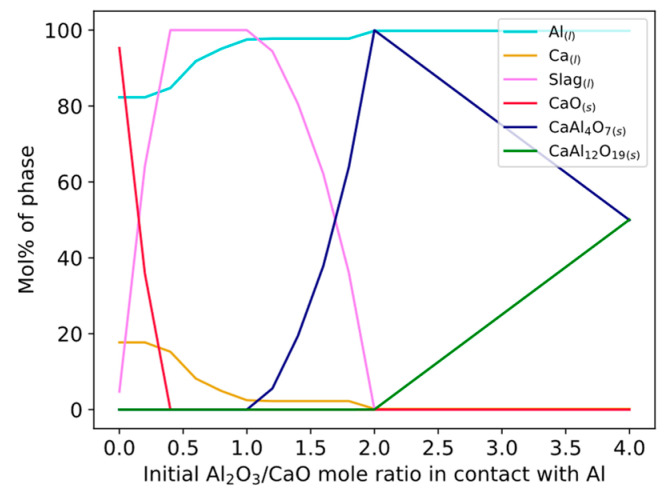
Phase formation between Al and CaO-Al_2_O_3_ slag for varying Al_2_O_3_/CaO ratios.

One or several of the reactions in Equations (12)–(14) is likely the main reaction for Ca production considering that Equations (3) and (4) do not occur spontaneously. The equilibrium calculations in Table 3 explain that CaAl_4_O_7_ was the most predominant phase at the Al alloy–slag interface for all times and temperatures. Equations (12)–(14) were calculated for varying Al_2_O_3_/CaO mole ratios (Figure 14), showing the relative number moles of CaAl_12_O_19_(s) and CaAl_4_O_7_(s). Experimental results showing CaAl_12_O_19_(s) suggest that this ratio is kept somewhat above 2 locally at the Al alloy–slag interface for all times at 1550 °C, for 3 min at 1600 °C, and perhaps shorter than 3 min for 1650 °C. The ratio decreases along with dissolution or reactions with Ca in Equations (15)–(17).

Reaction Equation (12) can impede the reaction rate due to the growth of CaAl_12_O_19_(s); however, metallic Ca can further reduce the calcium aluminate layers in steps according to Equations (15)–(17):

(15) $${\mathrm{Al}}_{2}O_{3}(s)+0.16\mathrm{Ca}(l)=0.16{\mathrm{CaAl}}_{12}O_{19}(s)+0.10\mathrm{Al}(l),\;∆G_{1600\;{}^{\circ}C}^{0}\;=\;-24.9\;kJ$$

(16) $${\mathrm{CaAl}}_{12}O_{19}(s)+1.71\mathrm{Ca}(l)=2.71{\mathrm{CaAl}}_{4}O_{7}(s)+1.14\mathrm{Al}(l),\;∆G_{1600\;{}^{\circ}C}^{0}\;=\;-248.3\;kJ$$

(17) $${\mathrm{CaAl}}_{4}O_{7}(s)+0.75\mathrm{Ca}(l)=1.75{\mathrm{CaAl}}_{2}O_{4}(l)+0.5\mathrm{Al}(l),\;∆G_{1600\;{}^{\circ}C}^{0}\;=\;-77.9\;kJ$$

where the melting point of Al_2_O_3_ is at 2054 °C. Reaction Equations (15)–(17) suggest that calcium serves as a catalyst, facilitating the transformation of the product layer from lower to higher oxidation states. Consequently, this catalytic activity mitigates the blocking effect of the product layer. The equilibrium calculations in Table 3, corresponding well with the measured Al alloy composition, indicate that an equilibrium is established where the Si-saturated Al alloy will promote the driving force for newly formed Si to instead disperse in the slag phase. Overall calculations show that the significance of the product layers is a function of the reaction with CaO and/or reduction by Ca. As CaAl_4_O_7_ was the major phase observed in experiments, the most probable rate-limiting reactions for the decrease in this phase are Equation (11) and/or Equation (17).

**Figure 15:**
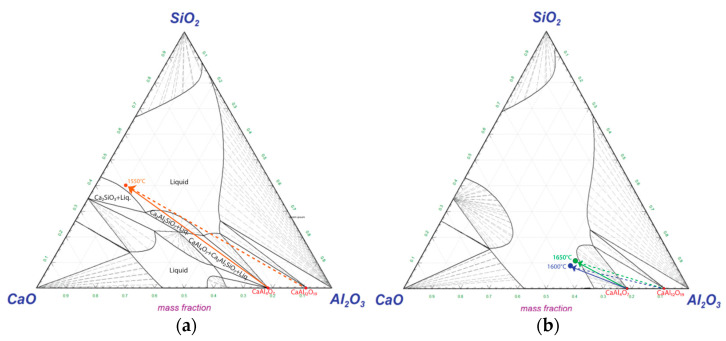
Dissolution paths at (**a**) 1550 and (**b**) 1600–1650 °C of interfacial calcium aluminate product layers in CaO-SiO_2_-Al_2_O_3_ during reaction with Al.

Hypothetical dissolution paths of CaAl_12_O_19_(s) and CaAl_4_O_7_(s) are drawn in Figure 15 with experimental compositions of the bulk slag in contact with the Si alloy droplets (here 3 min samples) corresponding to each temperature. Temperatures of 1600 °C and 1650 °C are drawn in a simulated diagram at 1600 °C as they are close to identical. The lines represent the expected change in composition at the Al alloy–slag interface. At 1550 °C a driving force for dissolution of Ca_2_Al_2_SiO_7_(s) phase exist and the driving force for dissolution of CaAl_12_O_19_(s) and CaAl_4_O_7_(s) is high (Figure 15a). At 1600–1650 °C, the driving force for the dissolution of CaAl_12_O_19_(s) and CaAl_4_O_7_(s) is less (Figure 15b).

The dissolution rate of alumina and calcium aluminates have been shown to be dependent on diffusivity and viscosity and controlled by mass transfer in the slag phase [35,36] and not dependent on the concentration gradient (represented by the length of lines in Figure 15). This suggests that dissolution is mainly favored by the increase in the relative CaO content.

In the present study, only a small concentration gradient (and overlapping margin of errors) was seen between the slag “outside” near the product layers and the slag bulk. This indicates that the diffusion of mainly oxygen and/or electrons or holes through the “equilibrium” bulk slag can be the rate-controlling step.

**Figure 16:**
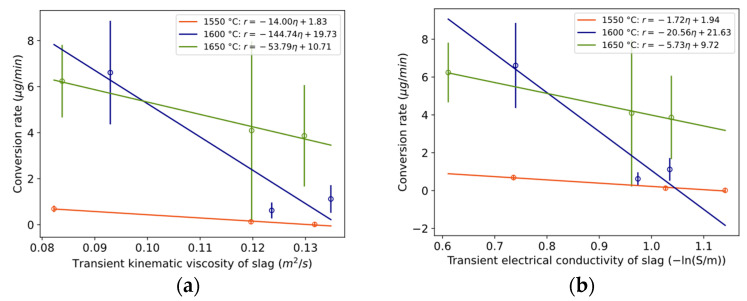
(**a**) Relationship between conversion rate and transient kinematic viscosity of slag with confidence interval [−20.6, −7.4], [−464, 175], and [−95.1, −12.4] at 90% confidence level and *p*-values 0.047, 0.21, and 0.077 for 1550, 1600, and 1650 °C, respectively. (**b**) Relationship between conversion rate and transient electrical conductivity with confidence interval [−3.0, −0.47], [−56.8, 15.7], and [−8.6, −2.8] at 90% confidence level and *p*-values 0.073, 0.17, and 0.051 for 1550, 1600, and 1650 °C, respectively. Whole vertical length of error bars equals two standard deviations.

**Figure 17:**
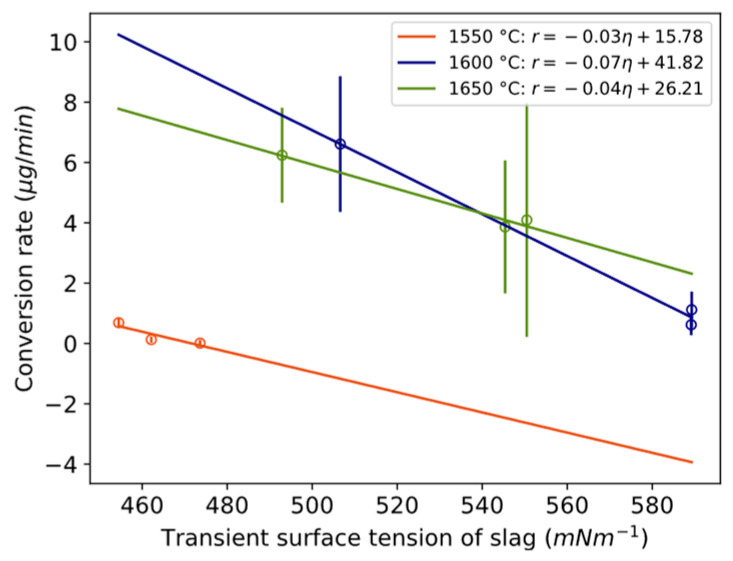
Relationship between conversion rate and transient surface tension of slag with confidence intervals [−0.25, 0.19], [−0.14, −0.0022], and [−0.13, 0.46] at 95% confidence level and *p*-values 0.30, 0.048, and 0.11 for 1550, 1600, and 1650 °C, respectively. Whole vertical length of error bars equals two standard deviations.

To assess whether conversion is dependent on mass transfer in the slag phase, the relationship between the conversion rate and physical properties is plotted in Figure 16a,b and Figure 17. Here, “transient” is defined as the average value during the compositional change during a reaction, between time periods 0 to 3 min, 3 to 20 min, and 20 to 40 min. The conversion rate statistically significantly decreases with increasing kinematic viscosity or electrical conductivity for 1550 °C and 1650 °C. It decreases with increased surface tension at 1600 °C, which according to Young’s equation is in a positive relationship with interfacial tension, here between the metal and slag. A statistically significant relationship was also valid for kinematic viscosity normalized by temperature for the temperature range 1600–1650 °C with confidence interval [−3.5, −0.33] at the 95% confidence level and *p*-value 0.03.

Despite the fact that interfacial tension was not measured, the following is an attempt to highlight its important contribution to the characteristic nature of the Al–slag reaction. Here, it is assumed that the shrinkage or flattening of the wetting droplet on the substrate is in a negative relationship with the apparent interfacial tension between the Al alloy and slag, recorded as “droplet shrinkage”. From Figure 9, it can be observed that the apparent interfacial tension initially decreases rapidly, followed by a slower decrease with time. It is claimed by several authors that the reaction itself, or the flow across the interface, is responsible for the decrease in interfacial tension, although it is described in the scope of different interfacial phenomena intervening in the process and is not yet well understood.

The major difference in droplet flattening is between 1550 °C and higher temperatures. The holding temperature of 1550 °C was the only temperature where the calcium aluminate product layer grew with time. The solutocapillary effect can be attributed to 1550 °C, where a higher oxygen potential in the slag relative to the metal initially leads to the mass transfer of oxygen from the slag to metal, and upon reaction, leads to increased oxygen content at the interface. At this temperature, the eddy currents produced from local difference in the degree of reaction along the interface are not strong enough to penetrate the product layer or reduce the mass transfer coefficients by blocking the diffusion.

This interfacial blockage can greatly be reduced by sufficient agitation [30]. Riboud and Lucas [23] and Gaye et al. [5] discussed that the oxide product layer may prevent the disappearance of interfacial tension phenomena. Rhamdhani et al. [32] studied the effect of Equation (2) in the Fe-CaO-SiO_2_-Al_2_O_3_ system on the interfacial change and showed that surface-active oxygen saturation at the metal–slag interface had a small but non-neglectable effect on interfacial tension depression. Work of Parra and Allibert [29] and Ooi et al. [25] also observed interfacial tension’s increase during interfacial product layer growth.

Riboud and Lucas [23] and Gaye et al. [5] showed that intense mass transfer across the interface, quantified to >0.1 g oxygen m^−2^s^−1^ due to the high driving force of, e.g., Equation (2), is responsible for interfacial tension decrease and emulsification of metal droplets given that the reaction is not impeded by product accumulation at the interface. Table 8 shows that the estimated flux in the present study surpasses this value, as can be expected due to the higher driving force in the present study. However, the impeded shrinkage of 1550 °C may agree with the statement that the product layer has a blocking effect.

### 4.2. Shrinking Behavior of Wetting Droplet

**Figure 18:**
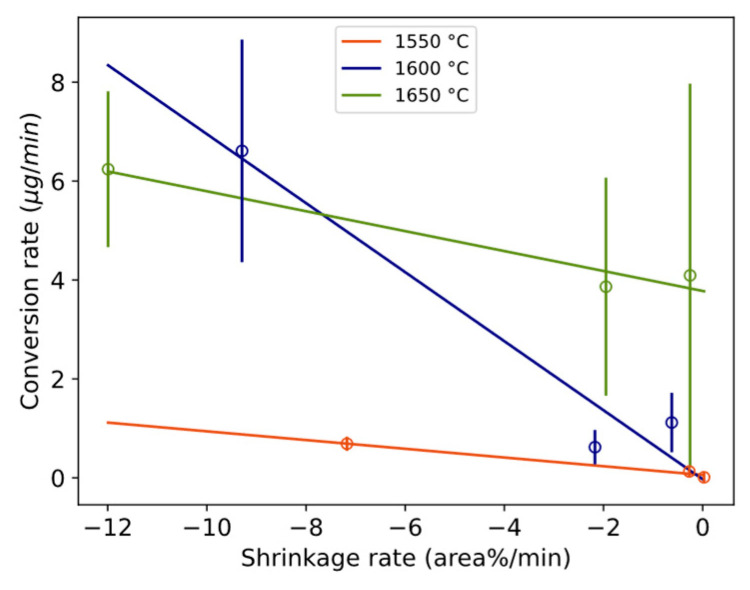
Relationship between conversion rate and the mean shrinkage rate of the wetting droplet with confidence intervals [−0.123, −0.054], [−1.231, −0.165], and [−0.341, −0.062] at 80% confidence level and *p*-values 0.08, 0.15, and 0.14 for 1550, 1600, and 1650 °C, respectively. Whole vertical length of error bars equals two standard deviations.

A statistical representation of the shrinkage or flattening rate of the wetting droplet and conversion rate is illustrated in Figure 18. The overall appearance reminds much of the corresponding Figure 16 and Figure 17 with viscosity/electrical conductivity, although here at a lower confidence level. The steeper slope for 1600 °C crosses 1650 °C and 1550 °C at higher and lower values, respectively. The relative size of the droplet decreases more in the first minutes compared to only slag, as shown in Figure 11. A similar statistical relationship was seen between the estimated oxygen flux across the interface (gm^−2^s^−1^) and the wetting droplet shrinkage rate. Whether the initial high conversion rate favors the shrinkage of the wetting droplet, the effect of physical properties of the slag on the apparent interfacial tension are evaluated.

The major change in the apparent interfacial interaction occurs in the slag as it melts and slowly spreads on the surface of the graphite substrate and simultaneously interacts with the metal. However, the inherent physical properties, viscosity, density, and surface, change with composition and temperature and therefore contribute to the observed wetting behavior. The spreading effect is a function of viscosity; hence, the apparent interfacial change should decrease with decreased viscosity. The viscosity [38] of the initial slag changes by −2, +29, and +22% from the time the liquid slag and Al had completely consolidated (1534 °C) to the 3 min holding time at 1550–1650 °C, respectively. From 3 min to 40 min, the change was +31%, +15%, and −17% for 1550, 1600, and 1650 °C, respectively.

The density of a liquid is a function of temperature and composition. An increase in slag density should be seen as a decrease in apparent interfacial change. The calculated density [39] of the initial slag decreases 3–4% from the time the liquid slag and Al had completely consolidated (1534 °C) to the target temperature of 1550–1650 °C. Upon reaction after 3 min, the density was 0% and decreases 10–12% for 1550 °C and 1600–1650 °C, respectively, with 1534 °C as a reference. From 3 min to 40 min, the change was only +1%, −2%, and 0% for 1550, 1600, and 1650 °C, respectively. Density, therefore, contributes significantly to the apparent interfacial change (Figure 10) only for trials at 1600–1650 °C during the first minutes of contact. In addition, the densities of formed solids CaAl_4_O_7_ and CaAl_12_O_19_ are higher and should, therefore, somewhat impede the decrease.

Surface and interfacial tension change with temperature and composition and both should decrease with decreased apparent interfacial change. The calculated surface tension of the initial liquid slag [39] changed from 487 mNm^−1^ at 1534 °C to 444 (1550 °C), 436 (1600 °C), and 426 (1600 °C), which are decreases of 9–12% depending on the target temperature. The interfacial tension is positively related to the surface tension of the two phases in contact. The interfacial tension between steel/Fe-C-Si-O and a similar slag in the present study increases with the CaO/SiO_2_ ratio [5,6], and Al_2_O_3_ has a somewhat decreasing effect above 10–20 wt.% Al_2_O_3_ [42]. In the present study, from the time the liquid slag and Al had completely consolidated (1534 °C) to the 3 min holding at 1550–1650 °C, the surface tension change was calculated as −4%, +19%, and +14%, respectively [39]. From 3 to 40 min, the changes were +5%, 0%, and +2%, respectively.

Up to the 3 min holding time, the change in viscosity, density, and surface tension together adds up to effects of −3%, +56%, and +56% on the shrinkage for 1550–1650 °C, respectively. Assuming they have an equal impact on shrinkage, and neglecting a potential effect of the solid oxide layer, physical properties of the slag have a strong impeding effect on the shrinkage of the droplet at 1600–1650 °C and a small positive effect on shrinkage at 1550 °C. This implies that the initial (0–3 min) sharp droplet shrinkage is not explained by the physical properties of the slag; hence, it is more likely explained by interfacial reaction phenomena.

For holding times of 3 to 40 min, the total effect was 37, 13, and −15%, respectively, and could partially explain the overall relative faster shrinkage at 1650 °C than 1600 °C. Even for longer holding times, the less shrinkage relative to only slag must be explained by interfacial tension depression. Riboud and Lucas [23] reported that interfacial tension recovers to high values after the reaction has calmed. This is consistent with the less shrinkage relative to only slag for 1600–1650 °C (Figure 11). Change to a high interfacial tension was also indicated by the spherical shape of the Al alloy and Si alloy droplets, i.e., surface energy minimization decreases the interfacial area of Si alloy droplets by coalescing and becoming spherical.

### 4.3. Proposed Phenomena Involved

It appears for the studied system that the decrease in the apparent interfacial tension is a result of interfacial reaction or fluid flow across the interface. The conductive characteristic of the current alloy–slag system makes the electrocapillary effect a possible candidate responsible for inducing gradients of surface and interfacial tension along the interface between the phases in contact. Richardson [30,31], Sharan [28], and Rhamdhani et al. [32] stated that these unequal local interfacial tensions induce a decrease in metal–slag interfacial tension and cause interfacial turbulence (Marangoni). The Marangoni flow-inducing increased interfacial area intuitively facilitates the global reaction rate. This will be referred to as the hydrodynamic interfacial phenomenon. As already discussed, the solutocapillary effect may be significant for 1550 °C. The electrocapillary effect and hydrodynamic phenomena are here discussed separately.

#### 4.3.1. Electrocapillary Effect

**Figure 19:**
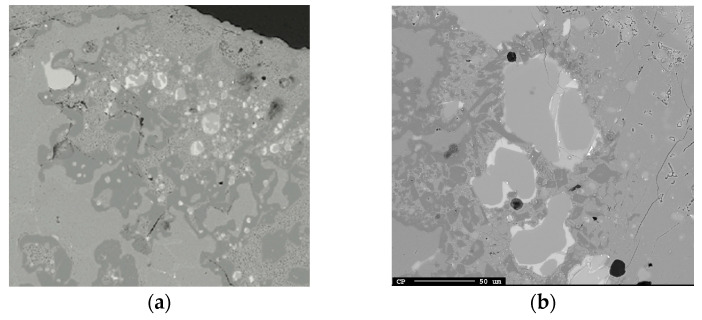
Examples of droplets near the Al alloy–slag interface at (**a**) 1600 °C and 20 min and (**b**) 1600 °C and 3 min.

**Figure 20:**
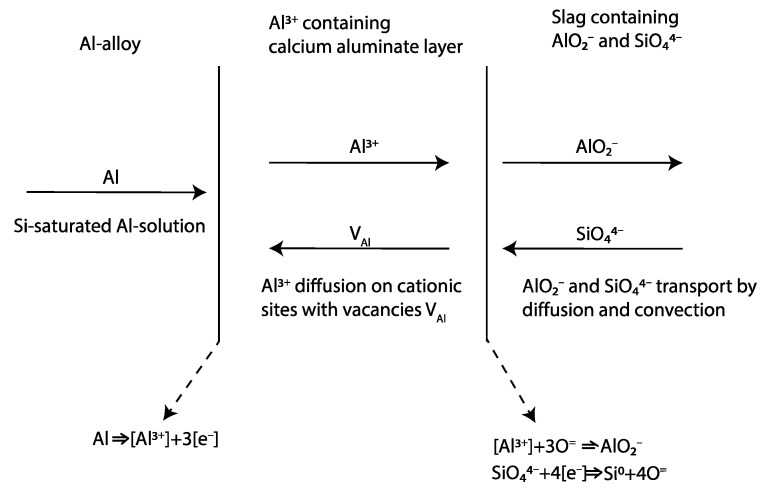
Oxido-reduction of SiO_2_ with Al across semi-permeable calcium aluminate layer allowing for the diffusion of Al^3+^ ions and charges (electrons or holes). Modified illustration from [29] by permission of the publisher (Taylor & Francis Ltd., http://www.tandfonline.com).

As Si alloy droplets were present in the slag, they also most likely are formed in slag or at the interface. Riboud and Lucas [23] referred emulsion to a “diffusion and stranding” mechanism, proposing that a chemical potential difference causes oxygen to strip from complex ions (Si_n_O_2n−x_)^2x−^ and migrate from the bulk slag regions rich in SiO_2_ towards the interface. Because Si is insoluble in slag, the Si precipitates on the spot in the slag. If the reaction rate is high enough and the mass transfer rate of species are different across the metal–slag interface, e.g., faster transfer of Al to the interface than Si from the interface, interfacial charge is developed. This is probable as the supply of Al is high in the Al alloy in direct contact and equilibrium with the product layer (Table 3) and the transport of Si (as Si droplets) and oxygen from/to the interface is relatively slow, as seen in Figure 19 A similar observation by Parra and Allibert [29] and Dumay and Allibert [34] was explained by the faster diffusion of Al from the metal to slag through the product layer than that corresponding to the Si from slag to the metal. This electrostatic force increases London forces or dipole interactions across the interface that decrease in interfacial tension [30,31]. Assuming that most of the Si alloy droplets are formed on the slag side of the alumina-rich layer, an illustration of the principal reaction that likely occurs is shown in Figure 20. Solvated electrons or holes, resulting from the oxidation of aluminum on the alloy’s surface, are capable of transporting charges essential for converting silica into metallic silicon within the slag. Consequently, an electrochemical reaction is feasible without the oxidizing and reducing agents being in direct contact.

Smaller Si alloy droplets closest to the Al alloy–slag interface than the corresponding wavelength of the perturbed Al alloy are seen in Figure 19a. In addition, some Si alloy droplets, containing some Ca, are located inside the calcium aluminate layer, indicating the slow transport of Si and AlO^2−^ relative to the oxido-reduction reaction rate. Relatively smaller Si alloy droplets are further away from the interface as seen in Figure 19b. This implies that Si can form both near or at the interfacial product layer or by the transport of electrons or holes and oxygen migration to/from the stripped silica ion complex to/from the interface.

**Figure 21:**
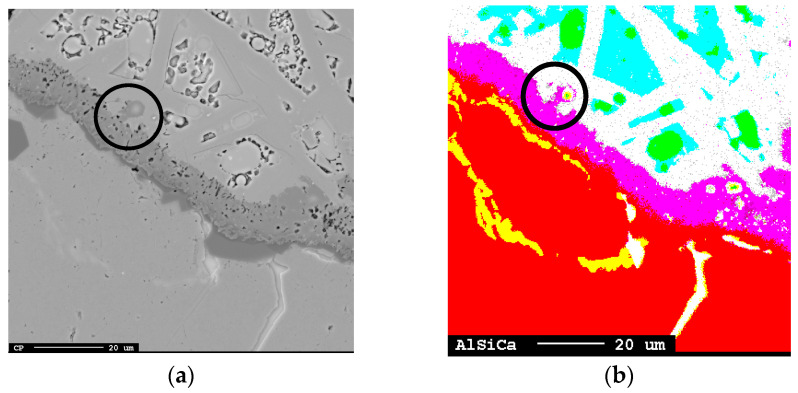
Al transport from the calcium aluminate layer to the liquid slag (marked) shown in (**a**) unmodified image and (**b**) modified mapping image, where red = Al, yellow = Al + Si, purple = calcium aluminate layer, green = Si, white/turquoise = CaO-SiO_2_-Al_2_O_3_ slag with turquoise having higher SiO_2_ content.

Simultaneously, as Si can form in the slag, Si in the Al alloy indicates that Si forms on the metal side. Table 3 indicates that the Al alloy is in equilibrium, or close to it, with the product layer. The driving force for diffusion from the product layer to the slag exists once the Al alloy has been saturated. The possible diffusion of Al from the solid calcium aluminate layer(s) to the liquid slag can be seen in Figure 21a,b, where the yellow represents the Al + Si phase and green represents pure Si. Further away from the interface, the pure Si phase (green) is seen surrounded by slag with the composition of the initial CaO-SiO_2_ slag indicating oxygen migration to the interface and electron or holes transport away from the interface to the bulk.

#### 4.3.2. Hydrodynamic Phenomena

**Figure 22:**
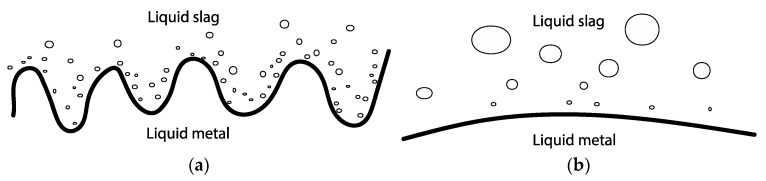
Emulsion of Si alloy droplets in slag due to (**a**) Kelvin–Helmholtz instability with an intense reaction rate and (**b**) emulsion and coalescence with a stable interface with a damped reaction rate.

Interfacial turbulence arises in reactions where spontaneous inequalities in surface tension develop across the interface at which the reaction occurs. Results suggest that interfacial turbulence due to natural convection or Marangoni flow occurred during the first minutes of contact between the Al and the slag. The hypothesis is proposed that interfacial reactions prompt Marangoni and/or natural convection at the Al alloy–slag interface, further promoting reaction. Following the work of Gopal [27], this flow at the interface consequently generates interfacial waves through a Kelvin–Helmholtz instability mechanism. These waves undergo growth and become unstable, and once culminated, lead to spontaneous emulsification of Al into slag, increasing the surface area and hence the reaction to form Si alloy, as depicted in Figure 22a. The Kelvin–Helmholtz instability phenomenon can be indicated by the cuplike shape of the lighter liquid (metal) [27]. Generally, it is possible that Kelvin–Helmholtz instability leads to the emulsion of both phases into one another, but in the present system, only metal emulsifies into slag. When the reaction rate dampens, emulsification reduces as the interface stabilizes and the droplets have time to coalesce, as seen in Figure 22b.

In the present study, the perturbed interface was seen as the surface of the Al alloy generally was significantly wavier for the shortest holding time (3 min) for all temperatures and longer times at 1550 °C. The latter is explained by solid product layer “freezing” the perturbed interface, although the reaction has dampened. At longer times at 1600–1650 °C, the surface was flattened and smoothed, corresponding to a high interfacial tension between the Al alloy and slag.

In addition, because Marangoni flow is a function of viscosity, it is proposed that the emulsion of metal in slag decreases with increased viscosity. This was found for a similar metal–slag system [26]. For the present system, it can be qualitatively observed in the work of Solbakk [20] for less viscous CaO-SiO_2_ slag with a decreasing CaO/SiO_2_ ratio. This agrees with the present study, shown in Figure 17, as the emulsion rate decreased with an increase in transient kinematic viscosity.

Rhamdhani et al. [32,43,44] and Zhao et al. [45], in their investigation of Al-alloyed Fe with CaO-SiO_2_-Al_2_O_3_, demonstrated that the augmented surface area resulting from the reaction promotes the overall reaction rate. Initially, the interfacial area experienced a sharp increase, reaching maximum values of up to 300–500%. This was followed by a gradual decrease as the reaction slowed down. This phenomenon was supported in the current study, wherein the initial perturbed interface led to the spontaneous emulsification of alloy droplets in the slag, subsequently transitioning to a smoother and more uniform interface of the Al alloy as the reaction progressed. Zhao et al. [45] noted that this phenomenon was mitigated in scenarios with a lower SiO_2_ content, attributed to a decrease in overall reaction determined from changes in the composition of both the metal and the slag. Sichen and White [46] also noted a transition in the dynamic fluctuation of the interfacial area, moving from high to low, in the examination of the interaction between MG-Si and CaO-SiO_2_-Al_2_O_3_ slag. The authors further put forward a theory applicable to the present work emphasizing the role of Gibbs energy to the phenomenon of emulsification and subsequently the re-coalescence of the emulsified droplets to the bulk metal. The authors propose that the processes of emulsification and recovery into the bulk metal depend not solely on the overall Gibbs energy variation between two states, but also on interfacial tension, generally expressed as the following:

(18) $$∆G_{\mathrm{Tot}}=∆G_{\mathrm{Reaction}\;}+\gamma \cdot A,$$

where $∆G_{\mathrm{Reaction}\;}$ is the Gibbs energy of a reaction, e.g., for Equation (2), and  γ·A is the interfacial term. Initially, the total Gibbs energy reaches its minimum because $∆G_{\mathrm{Reaction}\;}$ is highly negative, making it the dominant term. However, as the reaction progresses and approaches equilibrium, this term approaches zero. As the reaction degree reaches its maximum, emulsification also peaks. At this point, the system’s energy prefers to be minimized, leading to a significant decrease in the interfacial area, with the interfacial term becoming dominant.

**Figure 23:**
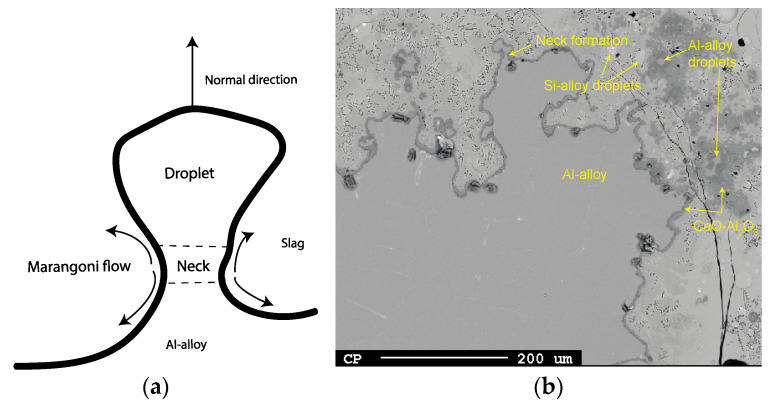
(**a**) Illustration of droplet detachment and (**b**) example image from EPMA (1550 °C, 3 min).

If the flux across the interface is sufficiently high, detachment of the droplet occurs after the necking formation at the Al alloy surface followed by an instant reaction, mainly by Equation (2). An example of this phenomenon is seen for 3 min at 1550 °C in Figure 23a,b. This was more difficult to find for 1600–1650 °C due to the faster reaction rate at these temperatures. According to Bainbridge and Sawistowski [33], Marangoni flow is induced by a concentration difference between the “droplet”, neck, and interface due to different degrees of reaction along the interface, hence leading to an interfacial tension difference along the metal–slag interface. Detachment occurs given that interfacial tension between the droplet and the slag is higher than for the neck and the slag.

During the initial period characterized by the highest conversion rates and consequent interfacial turbulence, droplets are expected to exhibit larger sizes due to droplet detachment, followed by the production of smaller droplets as the conversion rate slows. The extent of this mechanism’s influence can be assessed through examination of Figure 12 and Figure 13. The different trends observed between 1600 and 1650 °C for durations of 3–20 min can be attributed to the persistence of relatively high interfacial turbulence at 1600 °C during this timeframe, whereas at 1650 °C, the period of vigorous droplet detachment from interfacial turbulence concludes within 3 min, transitioning into a period of dampened but more consistent conversion with time. At 1550 °C, a trend akin to that at 1650 °C is observed, although it is attributed to a significantly lower coalescence rate relative to its conversion rate.

Overall, the size of Si alloy droplets is more related to coalescence (Figure 12 and Figure 13) during longer times rather than from droplet detachment due to interfacial turbulence during first holding minute(s). Consequently, the critical length of the necking for detaching must be relatively small in the range of 1–2 microns, i.e., considerably smaller than Si alloy droplets at 20–40 min at 1600–1650 °C.

Another sign of turbulence is the nonuniform shape of the Si alloy droplets at 3 min, different from spherical droplets at 20 and 40 min at 1600–1650 °C. In these cases, the Si alloy most often appears in the SiO_2_-richer slag region rather than surrounded by Al_2_O_3_ (Figure 2). Once the flow (mass transfer in slag) slows down (at 1600–1650 °C, 20–40 min), confirmed by the low conversion rate (Figure 6), the Si alloy minimizes surface energy by becoming spherical.

## 5. Conclusions

The interfacial phenomena influencing the aluminothermic reduction of a calcium silicate slag, which can either facilitate or impede reaction kinetics, were identified and discussed. The identification of phenomena impeding the reaction holds significant importance, thereby reinforcing the evidence advocating for the necessity of employing a well-stirred reactor. This underscores the crucial role of reactor design in optimizing reaction kinetics and overall process efficiency. In conclusion, the following findings emerge:

- Although no external convection was present, chemical reaction occurs instantaneously between Al and CaO-SiO_2_ at 1550–1650 °C. The estimated mass of emulsified Si alloy droplets and oxygen flux across the interface were highest during the first 3 min of the holding time, seemingly less significant at 1550 °C and somewhat higher at 1650 °C than at 1600 °C. The rate decreases significantly during the subsequent 3–20 min at all temperatures, statistically attributed to increased kinematic viscosity or decreased electrical conductivity at 1550 °C, and to increased surface tension at 1600 °C. A statistically significant relationship was observed at 1650 °C, similar to that observed at 1550 °C.
- On the surface of the Al alloy, a thin solid layer comprised of CaAl_12_O_19_ and CaAl_4_O_7_, measuring a few microns in thickness, was observed. Particularly notable was the presence of CaAl_4_O_7_, consistent across various temperatures and durations. This solid layer likely impeded the reaction at 1550 °C due to its slow dissolution, attributed to the partially liquid slag and comparatively higher viscosity compared to higher holding temperatures. At 1600–1650 °C, the complete dissolution of solids was observed between 3 and 20 min of the holding time.
- Novel characteristics found for this metal–slag system at higher temperatures of 1600–1650 °C compared to 1550 °C can be summarized as (i) a thinner calcium aluminate product layer, (ii) a higher conversion degree and conversion rate, (iii) more change in Al alloy and slag composition, (iv) faster shrinkage of the wetting droplet, and (v) the number per mass of Si alloy droplets was significantly less regardless of holding time. Trials at 1600 and 1650 °C appeared more similar in terms of overall reaction behavior, although deviation tended to increase above 3 min holding time due to more favorable liquid properties of the slag phase.
- Estimated oxygen flux (gm^−2^s^−1^) across the Al alloy–slag interface was well above the critical value (0.1) determined by previous work to obtain interfacial depression and emulsion. The flux was calculated to average 10.3–15.4 gm^−2^s^−1^ within 3 min of the holding time, followed by 3.6 gm^−2^s^−1^ between 3–20 min and 0.1 between 20–40 min at 1650 °C, correspondingly 0.7 and 1.7 for 1600 °C and 1.0 and 1.6 for 1550 °C.
- The electrocapillary effect and hydrodynamic phenomena represent potential occurrences during the reaction process. Apparent interfacial tension depression and Si alloy emulsion result from the accumulation of interfacial charge due to varying mass transfer coefficients of reactants and products, thereby inducing hydrodynamic phenomena that further facilitate the reaction. At 1550 °C, the presence of an interfacial product layer blocks weaker eddy currents, thereby impeding the reaction rate.
- This study underscores the significance of transient or product slag properties as the paramount parameter for controlling kinetics and facilitating metal–slag separation.

## Figures and Tables

**Table 1:** Initial slag composition (wt.%).

SiO_2_	CaO	Al_2_O_3_	Fe_2_O_3_	MgO	S
48.1	51.3	0.15	0.1	0.3	0.008

**Table 2:** Mass of Si alloy droplets per mapped area (µg/mm^2^) as a measure of conversion degree plotted with holding times for different temperatures. Error equals one standard deviation.

	3 min	20 min	40 min
**1550 °C**	2.1 ± 0.4	4.2 ± 1.8	4.5 ± 2.2
**1600 °C**	19.8 ± 6.7	30.5 ± 5.9	52.8 ± 12.0
**1650 °C**	18.7 ± 4.7	84.4 ± 37.5	166.2 ± 77.6

**Table 3:** Equilibrium calculation of compositions (wt.%) of Al alloy and calcium aluminate product layer.

Temp. (°C)	Time (min)	Meas. Al-Alloy			Equil. CaAl_4_O_7_	Equil. CaAl_12_O_19_	Equil. Al-Alloy		
		Al	Ca	Si			Al	Si	Ca
1550	3	95.5	3.4	0.05	97.3	2.7	96.2	3.4	0.33
1550	20	90.7	8.3	0.12	97.0	3.0	91.2	8.4	0.43
1550	40	86.2	12.5	0.08	95.5	4.5	86.9	12.6	0.54
1600	3	92.6	6.3	0.06	96.7	3.3	93.2	6.4	0.40
1600	20	88.2	10.5	0.08	95.9	4.1	88.9	10.6	0.50
1600	40	83.2	15.4	0.15	95.2	4.8	83.8	15.6	0.65
1650	3	93.6	4.8	0.24	98.6	1.4	94.7	4.9	0.39
1650	20	82.5	16.1	0.12	94.6	5.4	83.1	16.2	0.68
1650	40	81.6	17.3	0.05	93.5	6.5	81.8	17.5	0.73

**Table 4:** Concentration (wt.%) of product bulk slag in contact with Si alloy droplets. The uncertainty corresponds to one standard deviation. Error equals one standard deviation.

Al_2_O_3_	3 min	20 min	40 min
1550 °C	10.4 ± 12.5	16.6 ± 0.1	21.4 ± 0.2
1600 °C	53.0 ± 0.8	58.7 ± 3.4	55.7 ± 1.4
1650 °C	54.2 ± 1.2	50.1 ± 1.1	53.9 ± 6.4
**CaO**	**3 min**	**20 min**	**40 min**
1550 °C	48.7 ± 6.8	41.7 ± 3.2	45.02 ± 3.2
1600 °C	37.5 ± 2.2	36.9 ± 1.5	35.0 ± 1.8
1650 °C	33.7 ± 0.9	32.6 ± 2.2	36.3 ± 1.8
**SiO_2_**	**3 min**	**20 min**	**40 min**
1550 °C	40.4 ± 6.0	41.2 ± 3.3	33.1 ± 3.4
1600 °C	8.8 ± 3.0	3.8 ± 2.2	8.8 ± 3.1
1650 °C	11.5 ± 2.0	16.8 ± 3.4	9.3 ± 8.2

**Table 5:** Concentration difference (absolute wt.%) between slag right outside the calcium aluminates product layer and product bulk slag in contact with Si alloy droplets.

Al_2_O_3_	3 min	20 min	40 min
1550 °C	9.6	5.9	0.1
1600 °C	n.a.	1.5	5.1
1650 °C	2.7	11.1	5.9
**CaO**	**3 min**	**20 min**	**40 min**
1550 °C	−6.3	−4.6	−2.2
1600 °C	n.a.	−0.6	2.0
1650 °C	2.6	4.3	2.6
**SiO_2_**	**3 min**	**20 min**	**40 min**
1550 °C	−3.3	−1.3	2.0
1600 °C	n.a.	−1.2	−7.2
1650 °C	−5.5	−15.4	−8.3

n.a.: not available.

**Table 6:** Calculated global thermodynamic equilibrium (wt.%) using FactSage 8.1. Error equals one standard deviation.

	Si Alloy (wt.%)			Product Slag (wt.%)		
	Si	Al	Ca	SiO_2_	Al_2_O_3_	CaO
**1550 °C**	72.8 ± 0.02	4.77 ± 0.01	22.0 ± 0.01	7.3 ± 0.05	50.0 ± 0.06	42.4 ± 0.02
**1600 °C**	72.1 ± 0.02	5.4 ± 0.01	22.1 ± 0.01	7.6 ± 0.04	49.7 ± 0.06	42.4 ± 0.02
**1650 °C**	71.4 ± 0.02	6.0 ± 0.01	22.2 ± 0.01	8.0 ± 0.04	49.4 ± 0.06	42.4 ± 0.02

**Table 7:** The change in the number of Si alloy droplets per mass and time.

	0–3 min	3–20 min	20–40 min
**1550 °C**	+223.7	−25.9	−1.4
**1600 °C**	+28.6	−1.1	−1.4
**1650 °C**	+38.3	−5.7	−0.3

**Table 8:** Estimated oxygen flux (gm^−2^s^−1^) across the Al alloy–slag interface using the data in Figure 7, assuming spherical interfacial area, stoichiometry of Equation (2), and surface area factor of 2 equivalent to Riboud and Lucas [23].

	0–3 min	3–20 min	20–40 min
**1550 °C**	10.3	1.03	1.56
**1600 °C**	15.4	0.73	1.65
**1650 °C**	10.6	3.59	0.13

## Data Availability

Data are contained within the article and raw data is available upon request by contacting the corresponding author.
